# Continuity and utilization of health and community care in elderly patients with heart failure before and after hospitalization

**DOI:** 10.1186/s12877-018-0861-9

**Published:** 2018-08-13

**Authors:** Emma Säfström, Tiny Jaarsma, Anna Strömberg

**Affiliations:** 1 Sörmland County Council, Nyköping Hospital, Nyköping, Sweden; 2 Department of Medical and Health Sciences, Division of Nursing Science, Linköping University, Linköping, Sweden; 3 Centre for Clinical Research Sörmland, Uppsala University, Eskilstuna, Sweden; 4 Department of Social and Welfare Studies, Linköping University, Linköping, Sweden; 5 Department of Cardiology, Linköping University, Linköping, Sweden

**Keywords:** Heart failure, Health care utilization, Hospitalization, Continuity of care, Discharge, Elderly patients

## Abstract

**Background:**

The period after hospitalization due to deteriorated heart failure (HF) is characterized as a time of high generalized risk. The transition from hospital to home is often problematic due to insufficient coordination of care, leading to a fragmentation of care rather than a seamless continuum of care. The aim was to describe health and community care utilization prior to and 30 days after hospitalization, and the continuity of care in patients hospitalized due to de novo or deteriorated HF from the patients’ perspective and from a medical chart review.

**Methods:**

This was a cross-sectional study with consecutive inclusion of patients hospitalized at a county hospital in Sweden due to deteriorated HF during 2014. Data were collected by structured telephone interviews and medical chart review and analyzed with the Spearman’s rank correlation coefficient and Chi square. A *P* value of 0.05 was considered significant.

**Results:**

A total of 121 patients were included in the study, mean age 82.5 (±6.8) and 49% were women. Half of the patients had not visited any health care facility during the month prior to the index hospital admission, and 79% of the patients visited the emergency room (ER) without a referral. Among these elderly patients, a total of 40% received assistance at home prior to hospitalization and 52% after discharge. A total of 86% received written discharge information, one third felt insecure after hospitalization and lacked knowledge of which health care provider to consult with and contact in the event of deterioration or complications. Health care utilization increased significantly after hospitalization.

**Conclusion:**

Most patients had not visited any health care facility within 30 days before hospitalization. Health care utilization increased significantly after hospitalization. Flaws in the continuity of care were found; even though most patients received written information at discharge, one third of the patients lacked knowledge about which health care provider to contact in the event of deterioration and felt insecure at home after discharge.

**Electronic supplementary material:**

The online version of this article (10.1186/s12877-018-0861-9) contains supplementary material, which is available to authorized users.

## Background

Heart failure (HF) it is the end stage of several cardiac diseases, and an increasing number of people are diagnosed with HF worldwide [1]. In developed countries the prevalence of HF is estimated to be 1–2% [2] and increases with age. The mean age of patients diagnosed with HF is 77 years, and in the population over the age of 80 years the prevalence is 20% [3]. Patients with HF often suffer from multiple illnesses leading to polypharmacy and frailty [4]. Heart failure is characterized by alternating periods of clinical stability and instability. Periods of deterioration have serious consequences in terms of increased mortality and morbidity as well as great suffering for the individuals, and may result in patients needing hospitalization [5]. The patients’ situation after hospitalization is complex and it is difficult for them to get the overall picture without comprehensive context-oriented discharge planning [6]. The period post-discharge after hospitalization is characterized as a time of high generalized risk and instability [7]. Readmission rates are high after hospitalization due to HF deterioration, with about one quarter of patients being readmitted within one month [5]. The HF patients often receive care from multiple providers and facilities; thus, there is a potential danger of fragmentation of care [4]. Patients with HF have been found to visit the emergency room (ER), outpatient clinic and/or primary care multiple times every year [8–10] and 25% of the HF patients receive home care after hospitalization [11]. To reduce fragmentation, patients with HF need a seamless chain of care across hospital and primary care. This can only be achieved through close collaboration between the healthcare providers so that the follow-up and management of every patient is optimal and integrated [7, 12]. A seamless continuity of care is most at risk during the patients’ transition from an institutional care setting to the home [13].

A few previous studies have reported health care utilization [8–10] for patients with HF, and community care utilization is occasionally described [11, 14] but no studies have been found that describe both health and community care in HF patients in the period associated with hospitalization. Community care includes assistance with housekeeping, personal hygiene and/or dressing. Home health care includes health care provided by registered nurses. Furthermore, the American Heart Association (AHA) recognizes the lack of evidence for best practice of transition from hospital to home in HF patients, advocating further research to optimize the discharge process and the transition from one setting to another [4]. To reduce the fragmentation and make the HF care follow a better continuum between different caregivers, we need more insights into the HF patient’s own perspective on the journey through the community and health care system. The objectives were to describe, from the patients’ perspective and from a medical chart review, health and community care utilization prior to and 30 days after hospitalization, and the continuity of care in patients hospitalized due to de novo or deteriorated HF.

## Method

### Design and study setting

In this cross-sectional study, data were collected by structured standardized telephone interviews with patients and from their medical charts. The study was conducted at a district county hospital in central Sweden with approximately 120 hospital beds. The hospital had no specialized cardiology ward, so patients with HF were cared for in a general medicine ward. According to the hospital routine all patients had to receive written discharge information when discharged from the medical ward. The discharge information should include: information on diagnosis, medical treatment and exams performed during the hospitalization, changes in medication, and a plan for follow-up. A total of seven different primary care centers were located within the hospital catchment area. Most of them had a specially trained HF nurse during the study period. Elderly, fragile patients within the hospital catchment area may also be assisted in their home by community care and/or home health care. Community care included assistance with housekeeping, personal hygiene and/or dressing. Home health care included health care provided by registered nurses. During the time of the study, HF care could also be carried out by the mobile home care team where registered nurses and nurse assistants worked during the daytime, seven days a week. The mobile home care team was hospital-based and had resources to monitor patients in their homes and provide diuretics intravenously when needed.

### Study participants

This study enrolled patients hospitalized due to an episode of de novo HF or with deteriorating HF (ICD: I50.0, I50.1, I50.9, I42.0) as the primary cause of admission, or patients who developed significant HF symptoms during hospitalization for another primary diagnosis. Exclusion criteria were dementia, non-Swedish speaking, short anticipated survival, not answering the telephone, or discharge to nursing home.

### Procedure

Consecutive inclusion was carried out from January to June 2014 and from August to December 2014. A list of patients discharged from the medical wards was reviewed four times a week. Eligible patients were contacted by telephone by the first author within one week after discharge. If the patient did not answer the telephone after three calls, no further attempts were made. The patients were given verbal information about the study, and if they gave verbal consent, the phone call continued with the structured standardized interview. After the interview, additional data were collected from the medical chart. A review of health care utilization was conducted 30 days after discharge.

### Instruments

**Fig. 1:**
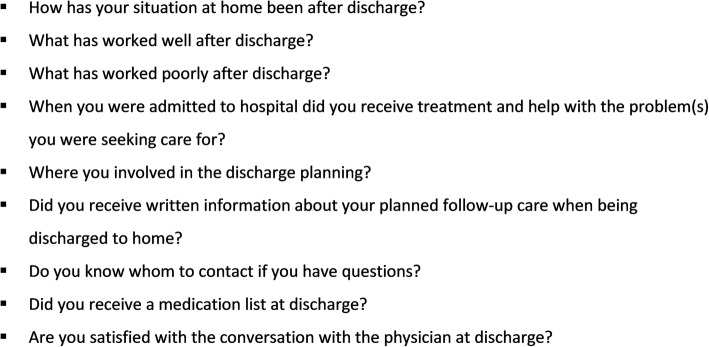
Questions from SALAR reported in the article. The questions are translated by the authors and are available in Swedish via the website http://www.webbkollen.com/ [15]

A questionnaire with 20 items addressing the time after discharge from hospital was used in the study [15]. The questionnaire was developed by The Swedish Association of Local Authorities and Regions (SALAR). The nine questions reported in this article are presented in Fig. 1. A second questionnaire was also used with items on symptoms before admission, reasons for admission and time of patient delay (Additional file 1). This questionnaire was developed by a research group in cardiovascular nursing research in collaboration with a patient representative and tested for face validity with a group of HF nurses and cardiologists [16]. Furthermore, the charts were reviewed by the first author for sociodemographic and clinical variables including multimorbidity as well as pharmacological treatment at admission and discharge. Multimorbidity was defined as co-occurrence of medical conditions and included diseases classified as etiology [17]. Renal failure was defined as ICD-10 code N17-N19. Assistance at home prior to and after hospitalization was listed as community care or home health care or assistance by the mobile home care team. Patient delay, which was defined as ‘the time between first symptoms and hospital admission’ was categorized in four different groups: one day, <one week, 1–2 weeks, > two weeks.

### Ethical issues

The study was designed and conducted in accordance with the principles of the World Medical Association Declaration of Helsinki [18]. Permission was granted by the Regional Ethical Review Board. All included patients were given verbal information regarding the study and gave verbal informed consent. It was underlined that participation was voluntary, could be terminated at any time without justification, and that not participating would in no way affect the patients’ future care. The patients were guaranteed confidentiality.

### Data analysis

Statistical analyses were performed using SPSS version 22.0. The characteristics of enrolled patients are presented as frequency and percentages for categorical variables and by mean and standard derivations for continuous variables. Spearman’s rank correlation coefficient was used for analysis of correlations between patient delay and symptoms on admission. A chi square test was used to compare health care utilization before and after the index hospitalization. A *P* value of 0.05 was considered significant.

## Results

### Demographic characteristics

A total of 370 patients (49% women, mean age 79 ± 10.7) were hospitalized due to HF during the inclusion period. A total of 249 patients were not eligible for inclusion. The main reasons for exclusion were not answering the telephone or being discharged to a nursing home. In total, 121 patients were included in the study, mean age 82.5 (±6.8) and 49% women. The ejection fraction (EF) was assessed by echocardiography in 59% of the patients. Within this group of patients, 25% had preserved EF and 75% had reduced EF (mean EF 36 ± 11). The demographical and clinical variables of the enrolled patients are presented in Table 1. The mean number of co-morbidities, besides HF was 2.9 (± 1.3), with cardiac co-morbidities such as hypertension (63%) and previous myocardial infarction (MI) and/or ischemic heart disease (51%) being the most common. The most frequent symptoms at admission were breathlessness (64%) and fatigue (36%) (Table 2). The median length of stay was four days and the median number of hospitalizations within six months prior to the index hospitalization was one, ranging from one to eight.

**Table 1:** Sociodemographic and clinical characteristic of hospitalized heart failure patients (*n* = 121)

Characteristics	
Age in years, mean (SD)	82.5 (± 6.8)
Woman, n (%)	59 (49%)
Cohabiting with family or others, n (%)	62 (51%)
Co-morbidities	n (%)
Hypertension	76 (63%)
MI / ischemic heart disease	62 (51%)
Atrial fibrillation	59 (49%)
Diabetes without complications	26 (22%)
Stroke	24 (20%)
COPD	20 (17%)
Renal failure	18 (15%)
Malignancy	16 (13%)
Other	14 (12%)
Period of time since diagnosed	n (%)
< 1 year	44 (36%)
1–5 years	33 (27%)
> 5 years	38 (31%)
Pharmacological treatment at discharge	n (%)
ACEI/ARB	91 (75%)
MRA	34 (28%)
Beta-blocker	100 (83%)
Total number of medication at discharge mean (SD)	11.58 (± 4.37)

*MI* myocardial infarction, *COPD* chronic obstructive pulmonary disease, *ACEI*; Angiotensin converting enzyme inhibitor, *ARB* Angiotensin receptor blocker, *MRA* Mineralocorticoid receptor antagonist

**Table 2:** Symptoms at admission in hospitalized heart failure patients (n = 121)

Symptoms	n (%)
Breathlessness	77 (64%)
Fatigue	44 (36%)
Chest pain	38 (31%)
Leg edema	35 (29%)
Orthopnea	30 (25%)
Cough	20 (17%)
Weight gain	13 (11%)
Dizziness	12 (10%)
Nausea	10 (8%)
Palpation	7 (6%)
Pulmonary edema	7 (6%)
Abdominal edema	2 (2%)

### Health care and community care utilization prior to the index hospital admission

Most of the patients had not visited any health care facility the month prior to the index hospital admission (Table 3) and some patients had visited several different health care facilities. A total of 7% of the patients had visited the ER without being admitted. A total of 40% of the patients had assistance at home from community care or home health care prior to index hospital admission. At the visit to the ER that ended with the index hospital admission, only 21% of the patients had a note of referral.

**Table 3:** Assistance at home and health care utilization 30 days prior and 30 days after the index hospitalization for patients with heart failure (n = 121)

Assistance at home prior index hospitalization	n (%)
Community care prior hospitalization	43 (35%)
Home health care prior hospitalization	22 (18%)
Mobile home care team prior hospitalization	5 (4%)
Assistance at home after index hospitalization	n (%)
Community care after hospitalization	56 (46%)
Home health after hospitalization	29 (24%)
Mobile home care team after hospitalization	22 (18%)
Health care facility visits 30 days prior to index hospitalization	n (%)
No prior visits to health care facility	62 (52%)
Primary care	34 (29%)
Hospitalized	16 (13%)
ER (without being admitted)	8 (7%)
Internal medicine outpatient clinic	3 (3%)
Health care facility visits 30 days after index hospitalization	n (%)
Primary care	27 (22%)
Rehospitalized	28 (23%)
ER (without being admitted)	19 (16%)
Internal medicine outpatient clinic	18 (15%)

*ER* Emergency room

A total of 33% of the patients were admitted on the same day of symptom onset, 33% within one week, 9% within two weeks and 24% delayed for more than two weeks from symptom onset. There were significant correlations between being admitted within the first week of symptom onset and prior MI and four typical symptoms of HF (Table 4). According to correlation analyses, patient delay was shorter when the patient experienced acute symptoms and signs such as chest pain and pulmonary edema, and longer when having symptoms of leg edema and fatigue. No statistically significant correlation was found between patient delay and age or sex.

**Table 4:** Factors significantly correlated with being admitted within the first week of symptom onset in patients hospitalized due to heart failure (n = 121)

Prior MI, rho (p)	0.275	(0.003)
Chest pain, rho (p)	0.214	(0.020)
Pulmonary edema, rho (p)	0.206	(0.025)
Total number of symptoms, rho (p)	- 0.199	(0.031)
Fatigue, rho (p)	- 0.203	(0.027)
Leg edema, rho (p)	−0.204	(0.026)

### Continuity of care

The total number of patients who received assistance at home from community care or home health care increased from 40% at admission to 52% after hospitalization. Prior to hospitalization, 35% of the patients received assistance from community care, increasing to 46% after discharge. Patients receiving assistance from home health care increased from 18% at admission to 24% after discharge. The number of patients receiving assistance from the mobile home care team increased from 4% prior to hospitalization to 18% after discharge (Table 3).

During the telephone interview within one week after discharge, half of the patients described their situation at home after discharge as functioning well, 29% reported their situation as both good and bad, and 20% said that their situation at home was functioning poorly. In total 50% of the patients experienced difficulties after discharge, most often due to burdensome symptoms of HF such as fatigue and dyspnea (Table 5). Difficulties were also due to medications, e.g. not having received necessary prescriptions or not being able to understand the list of medications. Two thirds of the patients stated they had participated in the planning of their discharge and 57% were satisfied with the discharge conversation. A total of 86% reported having received written discharge information and 89% had received a list of their medications. Two-thirds of the patients reported that they had knowledge of which health care provider to consult in case of deterioration or complications. Two thirds reported feeling safe and secure with their current health care and community care contacts.

**Table 5:** Concerns and symptoms in patients with heart failure after discharge (n = 121)

Symptoms	n (%)
Fatigue	38 (31%)
Shortness of breath	15 (12%)
Dizzy	7 (6%)
Lack of appetite	5 (4%)
Concerns	n (%)
Concerns regarding the medications	13 (11%)
Not enough community care after discharge	5 (4%)
The need of assistive equipment not met	4 (3%)
Not enough home health care after discharge	1 (1%)
Overburdened relative	1 (1%)

**Fig. 2:**
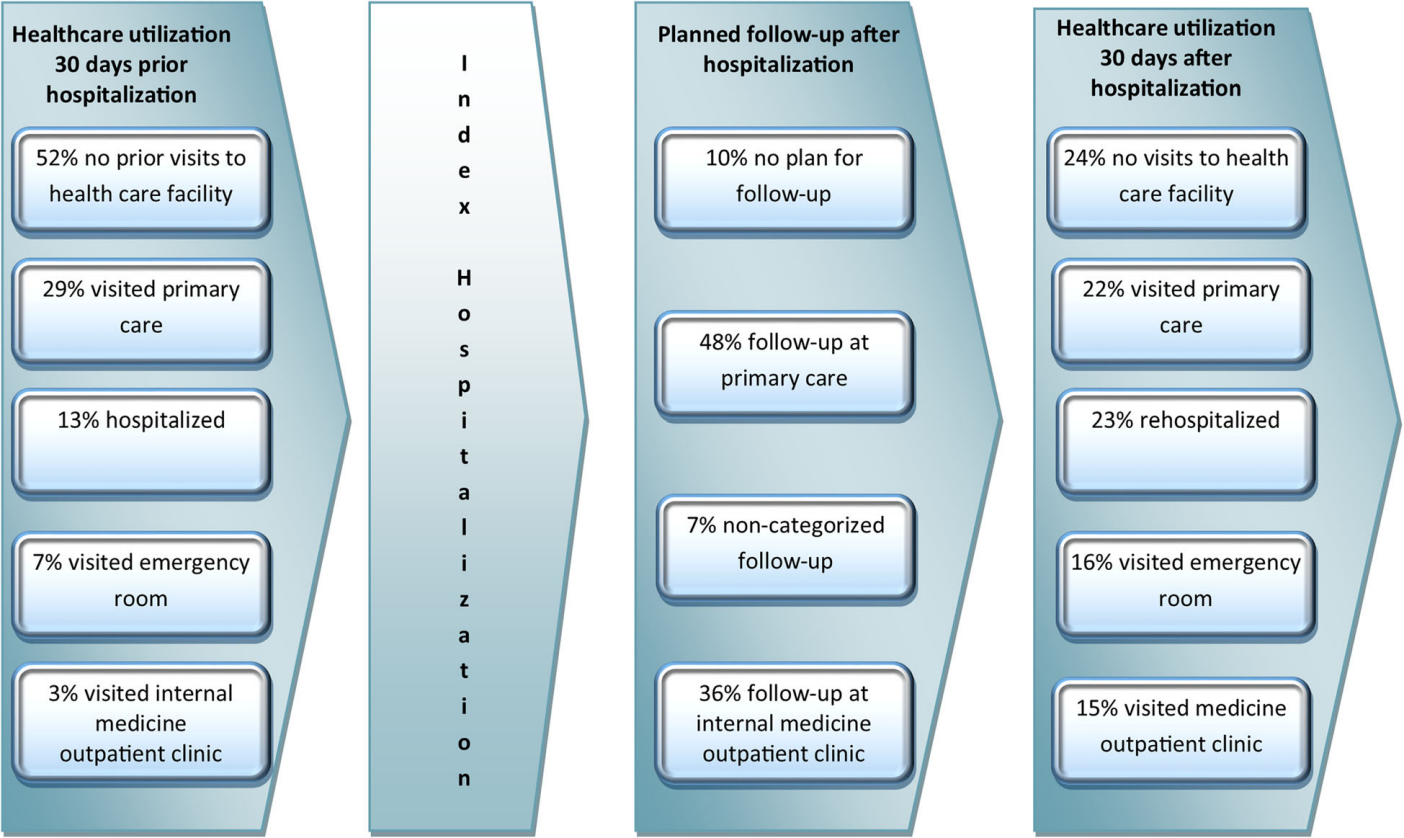
Health care utilization prior to and post-discharge by patients hospitalized due to heart failure (n 121)

Health care utilization prior to and post-discharge is presented in fig. 2. At discharge, 10% of the patients had no documented plan for follow-up, 48% were referred to the primary care (57% women, mean age 83.8 ± 6.7) and 36% to the outpatient medical clinic (47% women, mean age 80.7 ± 6.7). Seven percent had non-categorized types of follow-up; by telephone calls or at other outpatient clinics. Five patients were referred for follow-up to both the primary care and an outpatient clinic.

### Readmissions and health care utilization after discharge

A review of health care utilization 30 days post-discharge revealed a significant increase of health care utilization after hospitalization. Only 35% of the patients had visited a health care facility within one month prior to index hospitalization, and 55% of the patients had visited a health care facility within 30 days after hospitalization (p 0.002). Within 30 days after the index hospitalization, 22% of the patients had visited the primary care, 16% had visited the ER without being admitted, and 15% had visited the internal medicine outpatient clinic. A total of 18% had been visited by the mobile home care team. In total, 23% of the patients were readmitted within 30 days (Table 3). Most patients were readmitted due to HF or other cardiovascular problems as their primary or secondary diagnosis; only three patients were readmitted due to a condition not related to HF. Two patients came to the ER with a note of referral and the rest sought care on their own initiative. The mean time to readmission was 13 (±9) days. The most common symptoms at readmission were fatigue (40%), breathlessness (39%) and weight gain (17%). Among patients with planned follow-up in primary care, one in four (26%) had visited the primary care within 30 days after discharge. In patients with planned follow-up in outpatient internal medicine clinic, 36% had visited the outpatient medicine clinic within 30 days after discharge.

## Discussion and conclusion

### Discussion

This cross-sectional study, combining the perspective of elderly patients as well as data from the medical charts on health and community care, revealed novel aspects of the continuity of care in patients hospitalized due to HF. The aspects included patients’ care seeking, health and community care utilization, as well as patients’ experiences of continuity of care. All these aspects need to be further addressed in order to improve the HF care.

A majority of studies on HF patients have exclusion criteria based on comorbidity, and 25% apply an upper age limit [19], leading to underrepresentation of the HF population [20]. The high mean age in our study highlights the situation for elderly patients burdened with multimorbidity.

It was striking to see that most patients turned to the ER in the first instance when their condition deteriorated and not to the primary care center or outpatient HF clinic in which they were enlisted. The number of patients who came to the ER without referral have increased from 62% in 1999 [21] to 79% in this present study. The ER should not be the first health care facility to contact when symptoms of deteriorated HF occur. This results in meeting a substantial number of different physicians and nurses over time, which may contribute to flaws in the continuity of care so that patients experience the care as fragmented [22]. Horowitz et al. found that patients preferred to contact the ER instead of their primary care, when treatment was immediately available at the ER and the accessibility of primary care was perceived to be low [23]. Low accessibility of primary care is confirmed in our study, when only 26% of the patients with a planned follow at primary care had actually visited the primary care within 30 days after discharge.

Among the patients enrolled in this study, 86% of them recalled receiving written information at discharge, but only two-thirds knew which health care provider to consult when deterioration or complications occurred. Similar flaws in the continuity of care were found in a study from 2008 where 30% of the patients reported no knowledge of this key information [24]. This either implies that the discharge information lacked this important information, or that the patients were unable to assimilate the information. Prior trials reveal coherent communication behavior as an essential factor in the discharge process [13, 25], and lack of proper discharge information has been found to be a contributing factor to readmissions [25]. Since the HF patients are often old and fragile it is even more important to ensure that information given is correctly understood [26]. It has previously been found that patients sometimes do not read the information they receive [27] which further emphasizes the importance of the use of techniques such as teach-back during discharge conversation [28]. Both the European Society of Cardiology (ESC) and AHA state that discharge planning is crucial to secure the continuity of care. As a part of comprehensive discharge planning, the patient should be provided with information on sufficient self-care behavior and a detailed plan for follow-up, which should include facilitated access to care [2, 4]. The ESC even suggests that HF patients are not medically fit for discharge if they have not been provided with tailored education [2]. For old and cognitively impaired patients it is important not only to provide information adjusted to their preferences and cognitive ability, but also provide to teach skills in how to manage self-care and assess the need of support from caregivers and community services [29, 30].

Prior studies suggest that the patient delay may be due to difficulties in recognizing symptoms of decompensated HF, and that the patients use a “wait and see” mentality [31, 32]. However, insights from this study also reveal that one third of the patients did not know which health care provider to contact if they had questions or deterioration occurred, and this uncertainty may be a contributing factor to patient delay.

#### Follow-up in primary care

The proportion of patients referred for follow-up in primary care and at the internal medicine outpatient clinic respectively has been about the same for the last 20 years. In 1995, 57% of the patients were referred to the primary care [21], and 48% of the patients enrolled in this study were referred to the primary care. The patient preference seems to be follow-up at internal medicine outpatient clinics. Two recent studies on follow-up found that patients declined study participation due to the risk of being assigned follow-up at primary care [12, 33]. However, these days, when the competence of primary care has been enhanced with HF nurses and the quality of the care can be ensured [34, 35], it would be advantageous and cost-effective to refer stable HF patients who are on optimal dosing of medicines to the primary care, which complies with the guidelines from ESC [2]. Furthermore, guideline adherence and patient adherence to medication have been found to be maintained when follow-up is managed within primary care [12, 36]. The availability of primary care is too low [37], and only one fourth of the patients with planned follow-up at primary care actually had a follow-up visit at primary care within 30 days post-discharge. The exact reason for this is unclear, but prior studies have found flaws in discharge communication and information transfer from hospital to primary care and lack of resources [38, 39], which could prolong the time from discharge to follow-up. Early follow-up, and follow-up with home visiting programs and multidisciplinary interventions have been found to reduce readmission and mortality [40].

#### Problems after discharge

Many of the patients still experienced troublesome symptoms after discharge. Fatigue and shortness of breath was frequently reported, and these were also the most common symptoms in patients readmitted within 30 days after the index hospitalization. Distressing symptoms have been described as a common reason for rehospitalization [41]. To still experience the same symptoms suffered on admission when discharged, might increase the feeling of uncertainty at home. Besides symptoms, concern regarding medication was a factor that made the situation at home bothersome for many of the patients (Table 5). This is in line with a recent review that found that medication-related difficulties after discharge are common in HF patients [42]. Since the goals of pharmacological treatment are relief of symptoms, to improve survival and decrease the need for hospital admission [2] it is elementary that patients are given the best preconditions to handle the pharmacological regime after discharge. Before discharge it should be ensured that the patients have all they need to manage the medical treatment [43]. Symptoms of deterioration, as well as medication-related problems are factors contributing to hospital readmission [25, 44]. The period after hospitalization is characterized as a time of high generalized risk [7], and health care utilization seemed to increase 30 days after hospitalization, when 55% of the patients had visited a health care facility. Some increase in health care utilization such as planned follow-up visits and home health care is a positive reflection of intensified attention to patient monitoring and follow-up after discharge and may improve outcomes. However, it must be considered as a flaw in HF care and a flaw in the continuity of care that a total of 35% of the patients were rehospitalized or visited the ER within the first month after discharge. Furthermore, it is deplorable that so few of the patients had their planned follow up visit at primary care or an outpatient clinic within the first month after discharge.

### Study limitations

There are potential limitations to the present findings. The number of patients was relatively small, and they were all treated at the same hospital. The health care facility system varies between counties, making generalization limited. Another weakness of this study is the fact that we did not retrieve data regarding whether the patients had contacted any health care facility or “Swedish Healthcare Direct 1177” by telephone. Only data on physical visits were collected and it is possible that the number of patients who had contacted health care facilities was higher. Another limitation is the fact that there was poor documentation in the medical records of the New York Heart Association (NYHA) functional classification in the medical charts. According to HF guidelines, NYHA classification should be used to describe the severity of symptoms and exercise intolerance and there is an association among NYHA class and hospitalization and death [2]. The NYHA classification would have been a useful variable in correlation analysis and it is possible that it might have influenced the results. Furthermore, the NYHA classification might be a variable that affects the patient’s experience of their situation at home after hospitalization.

#### Recommendations to overcome flaws in the continuity of care:

- Ensure that patient has understood the discharge information correctly.
- Discharge information should include contact information of an appropriate health care provider
- Ensure that discharge information or referral is available in primary care immediately after discharge
- Continuity of care should be a prioritized area of improvement work

## Conclusion

The findings of this study, describing the care utilization and continuity of care in the real-world elderly hospitalized HF patients, showed that most patients had not visited any health care facility during the month prior to the index hospital admission, and that health care utilization increased significantly after hospitalization. The number of patients who received assistance at home increased after hospitalization and patients were most often referred for follow-up in primary care. We also found that, although most patients received written information at discharge, many of them felt insecure after discharge and lacked knowledge about which health care provider to consult in the case of deterioration or complications.

## Additional file

Additional file 1: Questionnaire with items on symptoms before admission, reasons for admission and time of patient delay. A translated version of a questionnaire with items on symptoms before admission, reasons for admission and time of patient delay. The questionnaire was developed by a research group in cardiovascular nursing research in collaboration with a patient representative and tested for face validity with a group of HF nurses and cardiologists [16]. For permission to use the questionnaire, please contact Anna Strömberg: anna.stromberg@liu.se (DOCX 18 kb)

## Abbreviations

AHA: American Heart Association
EF: Ejection Fraction
ER: Emergency room
ESC: The European Society of Cardiology
HF: Heart failure
ICD: International Statistical Classification of Diseases and Related Health Problems
MI: Myocardial infarction
NYHA: New York Heart Association
