# Supplementary material for: Continuity and utilization of health and community care in elderly patients with heart failure before and after hospitalization
Source: BMC Geriatr. 2018 Aug 13;18:177. doi: 10.1186/s12877-018-0861-9 (PMC6090801; doi:10.1186/s12877-018-0861-9)
Supplement: Supplementary file 1 — Questionnaire with items on symptoms before admission, reasons for admission and time of patient delay. A translated version of a questionnaire with items on symptoms before admission, reasons for admission and time of patient delay. The questionnaire was developed by a research group in cardiovascular nursing research in collaboration with a patient representative and tested for face validity with a group of HF nurses and cardiologists [16]. For permission to use the questionnaire, please contact Anna Strömberg: anna.stromberg@liu.se (DOCX 18 kb) [file 12877_2018_861_MOESM1_ESM.docx]

# Questionnaire assessing factors influencing care seeking and reasons for admission

**Initiative for seeking care at the hospital**

- Own initiative
- Initiative from next of kin
- Initiative from community care staff
- Initiative from home health care nurse
- Referral from general practitioner

**The time between onset of first symptoms and hospital admission**

- one day
- < one week
- 1-2 weeks
- > two weeks

**Symptoms before admission**

- Breathlessness
- Orthopnea
- Pulmonary edema
- Leg edema
- Abdominal edema
- Cough
- Weight gain
- Fatigue
- Dizziness
- Palpation
- Chest pain
- Nausea
- Other

**Reason for heart failure deterioration**

- Acute myocardial infarction or angina
- Arrhythmia
- Hypertension – untreated or undertreated
- Non-adherence to treatment and/or self-care
- Suboptimal medical heart failure treatment
- Changes in medical treatment
- Other reasons e.g. emotional stress, excessive alcohol intake, pregnancy
- Complications due to other illness, infection, pulmonary embolism, liver disease, renal failure, thyrotoxicosis or anemia.
